# K-Ras and cyclooxygenase-2 coactivation augments intraductal papillary mucinous neoplasm and Notch1 mimicking human pancreas lesions

**DOI:** 10.1038/srep29455

**Published:** 2016-07-06

**Authors:** Sara Chiblak, Brigitte Steinbauer, Andrea Pohl-Arnold, Dagmar Kucher, Amir Abdollahi, Christian Schwager, Birgit Höft, Irene Esposito, Karin Müller-Decker

**Affiliations:** 1 Tumor Models, Deutsches Krebsforschungszentrum (DKFZ),69120 Heidelberg, Germany; 2 German Cancer Consortium (DKTK); Molecular & Translational Radiation Oncology, Heidelberg Ion Therapy Center (HIT), Heidelberg Institute of Radiation Oncology (HIRO), University of Heidelberg Medical School and National Center for Tumor Diseases (NCT), DKFZ, 69120 Heidelberg, Germany; 3 Birgit Höft, Division of Cancer Epidemiology, DKFZ, 69120 Heidelberg, Germany; 4 Institute of Pathology, Heinrich-Heine-University of Duesseldorf, 40225 Duesseldorf, Germany

## Abstract

Mutational activation of K-Ras is an initiating event of pancreatic ductal adenocarcinomas (PDAC) that may develop either from pancreatic intraepithelial neoplasia (PanIN) or intraductal papillary mucinous neoplasms (IPMN). Cyclooxygenase-2 (COX-2)-derived prostaglandin E_2_ (PGE_2_) is causally related to pancreatic carcinogenesis. Here, we deciphered the impact of COX-2, a key modulator of inflammation, in concert with active mutant K-Ras^G12D^ on tumor burden and gene expression signature using compound mutant mouse lines. Concomitant activation of COX-2 and K-Ras^G12D^ accelerated the progression of pancreatic intraepithelial lesions predominantly with a cystic papillary phenotype resembling human IPMN. Transcriptomes derived from laser capture microdissected preneoplastic lesions of single and compound mutants revealed a signature that was significantly enriched in Notch1 signaling components. *In vitro*, Notch1 signaling was COX-2-dependent. In line with these findings, human IPMN stratified into intestinal, gastric and pancreatobillary types displayed Notch1 immunosignals with high prevalence, especially in the gastric lesions. In conclusion, a yet unknown link between activated Ras, protumorigenic COX-2 and Notch1 in IPMN onset was unraveled.

Despite innovations in basic research, diagnostic imaging, and surgery, patients with invasive pancreatic ductal adenocarcinoma (PDAC) still have a poor prognosis with an overall 5-year-survival rate of about 5%, mainly due to metastasis at the time of diagnosis in a high percentage of patients and lack of efficient therapy modalities^1^.

PDAC may arise via multiple precursor sequences, the most prevalent pancreatic intraepithelial neoplasia (PanIN), the less frequent cystic intraductal papillary mucinous neoplasms (IPMN), the rare mucinous cystic neoplasms (MCN) and probably also from atypical flat lesions^2^. The PanIN-PDAC and IPMN-PDAC sequences are well characterized at morphological and genetic levels^3^,^4^. Recent whole exome sequencing studies verified the prominent incidence of K-Ras somatic mutations resulting in a constitutive activation of K-Ras protein with persistent downstream signaling in early PanINs and IPMN^5^,^6^. Actually, activating K-Ras mutations are key genetic alterations for initiation of PDAC along both PanIN as well as IPMN sequences^7^.

The P48Cre/LoxP-STOP-LoxP-(LSL) K-Ras^G12D^ conditional mouse model of PDAC, referred to in this study as PK line (instead of the usual model identifier KC), mimics well the human situation in clinical, histopathological, and molecular features. Under the control of pancreas-specific P48-promoter, the heterozygous K-Ras^G12D^ knock-in allele is expressed in embryonic pancreatic progenitor cells, in centroacinar as well as acinar cells in the adult. This mouse mutant develops murine PanIN with 100% penetrance^8^,^9^. However, only a subset of 3% to 5% of these mice develops PDAC beyond the age of one year, indicating that additional events are necessary for adenocarcinogenesis, also in the mouse.

Proinflammatory cyclooxygenase-2 (COX-2) is an established factor linking inflammation with cancer in various organs including pancreas^10^. In humans, aberrant COX-2 overexpression accompanies chronic pancreatitis^11^. Patients suffering from this disease have, as compared to the normal population, a 15-fold increased risk to develop pancreatic cancer^12^. Moreover, cerulein-induced chronic pancreatitis in adult mice, associated with aberrant COX-2 overexpression, is essential for the induction of classical PDAC by K-Ras^G12V^ oncogene^13^,^14^. As in many other epithelial tumor entities, up to 90% of human PDAC show up with aberrant COX-2 expression as compared to normal tissue^10^,^15^. We have previously shown that cytokeratin 5 promoter (K5)-driven overexpression of COX-2 (C transgenic line) resulted in preinvasive duct-derived neoplasms of pancreas^15^. The K5-promoter was shown to drive expression of cyan fluorescent protein in few cells of the duct epithelium obviously including single centroacinar cells^16^; data that is consistent with the immunohistochemical detection of CK5 in few cells of the pancreatic ducts^15^,^17^,^18^. Treatment of PK or C transgenic mouse lines with nonselective COX or selective COX-2 inhibitors respectively resulted in a reduced tumor load^15^,^19^.

Here, we show that concomitant activation of K-Ras and COX-2 signaling in a K5 COX-2. P48-Cre/K-Ras^G12D^ compound mutant (CPK line) accelerated development of PanIN and most importantly induced exclusively IPMN lesions. This was also accompanied by COX-2-dependent upregulation of the Notch1-Hes1 signaling pathway in mice, a finding that correlated to Notch1 expression in human IPMN, here significantly the gastric-type IPMN. Thus this newly recognized signaling axis in cooperation with oncogenic K-Ras is proposed to preferentially drive IPMN versus PanIN-associated PDAC.

## Results

To unravel the effect of proinflammatory and protumorigenic COX-2 onto K-Ras^G12D^-initiated pancreatic cells, the conditional compound mutant K5 COX-2/675^+/wt^.P48-Cre/K-Ras^G12D/wt^ (CPK) was successfully established for comparison with the P48-Cre/K-Ras^G12D/wt^ (PK), the group of C transgenic lines K5 COX-2/675^+/wt^ (C), K5 COX-2/675^+/wt^/P48Cre (CP), and K5 COX-2/675^+/wt^ /K-Ras^G12D/wt^ (CK) as well as a control group of wild-type (WT), P48-Cre (P) and K-Ras^G12D/wt^ (K) lines (SFig. 1). While the previously studied homozygous Tg(K5-COX-2/675^+/+^) mouse line on the NMRI outbred background showed up with pronounced epithelial dysplasia in skin epidermis, mammary gland, urinary bladder and pancreas^15^,^20^, the congenic heterozygous Tg(K5-COX/675^+/wt^) (C) mouse line on the C57BL/6N background used in this study displayed spontaneously less pronounced histological changes in pancreas when comparing 12-months-old transgenic and WT cohorts (SFig. 2).

### Enhanced formation of early stage precursor lesions of PDAC in CPK mice

**Figure 1:**
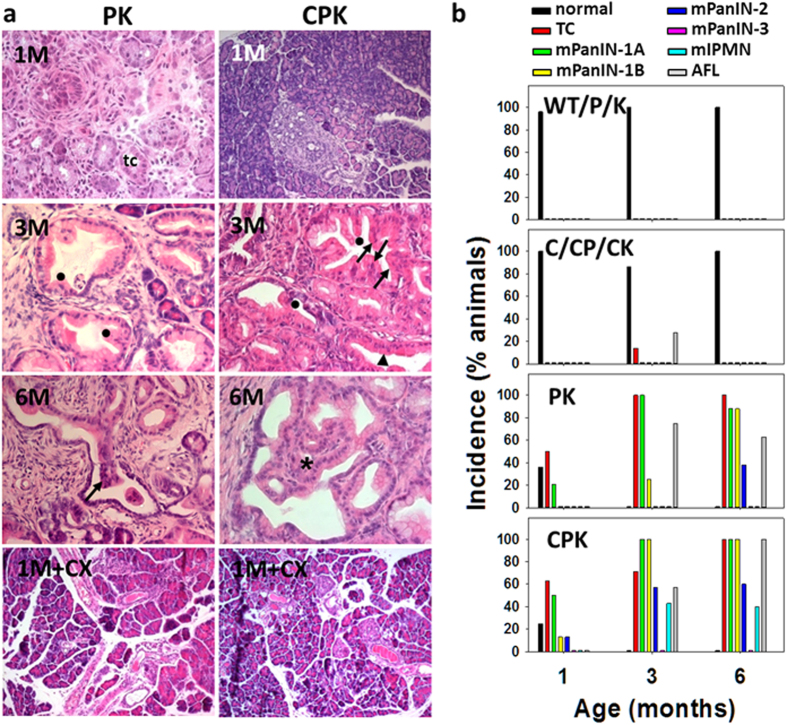
Histopathological analysis of pancreata of PK and CPK mice and incidence of dysplastic lesions at increasing age. **(a)** Representative pictures of HE-stained pancreatic sections at the ages of 1 month (1M), 3 months (3M), and 6 months (6M). (1M): PK mice developed tubular complexes (TC) and mucin-rich mPanIN-1A lesions. CPK mice developed multiple TC, mPanIN-1A and mPanIN-1B as well as mPanIN-2 lesions. (3M): PK mice displayed multiple TC and lesions graded mPanIN-1A and mPanIN-1B. CPK mice displayed multiple TC, mPanIN-1A, mPanIN-1B, and mPanIN-2. (6M): PK mice with multiple TC, mPanIN-1A, mPanIN-1B, and mPanIN-2 pancreatic lesions. CPK mice with multiple TC, mPanIN-1A, mPanIN-1B, mPanIN-2. Lesions start to acquire IPMN borderline features such as cellular budding into the lumen, and complete loss of cell polarity. (1M+CX): Lack of pathological changes in PK and CPK mice (n = 3, each) exposed to celebrex postnatally for 4 weeks. **(b)** Incidence of TC, mPanIN, and IPMN lesions in pancreata of PK and CPK mice as compared to C /CP/CK and WT/P/K mice of increasing age. Percentage of mice diagnosed with neoplastic pancreatic ducts by grade mPanIN-1A, mPanIN-1B, mPanIN-2, mPanIN-3, and IPMN-3 (borderline) as well as TC, and atypical flat lesions (AFL). Groups of 1M, 3M, and 6M-old mice of the indicated genotypes were analyzed. 1M: n = 25 WT/P/K, n = 19 C/CP/CK, n = 14 PK, n = 8 CPK; 3M: n = 5 WT/P/K, n = 7 C/CP/CK, n = 3 PK, n = 7 CPK; 6M: n = 15 WT/P/K, n = 4 C/CP/CK, n = 8 PK, n = 5 CPK. *Tubular complexes (TC), mPanIN-1A* (▲), *mPanIN-1B* (●)*, mPanIN-2* (↑), mIPMN-3 borderline (*). Magnifications: 10x (1M, 1M+CX); 40x (3M, 6M).

Histological analysis of mouse pancreata of increasing ages was performed from each genotype. Pancreata from 1 to 12-months-old mice with WT, P, K, C, CP, and CK genotypes showed a normal pancreatic phenotype as exemplified for the 12 months-old group (SFig. 3). Therefore in subsequent studies, mice of these genotypes were put into groups of C/CP/CK mice and WT/P/K mice for comparison with PK and CPK mice. In contrast, diagnostic examination of pancreatic sections from PK and CPK mice revealed prominent pathological changes (Fig. 1a). Tubular complexes (TC) were observed very frequently, the incidence of which increased in both genotypes with increasing age (Fig. 1b). At the age of 1 month (1M), about 20% of PK and 50% of CPK mice developed mucinous lesions reminiscent of murine (m) mPanIN-1A lesions. CPK mutants presented additionally with mPanIN-1B and mPanIN-2 at incidences of 12.5%, each. In the 3-month (3M)-old PK cohort, the incidence of mPanIN-1A, mPanIN-1B, and mPanIN-2 were 100%, 25%, and 0% respectively. However, at the age of 6 months (6M) an increasing fraction of PK presented with mPanIN-1A (>80%), mPanIN-1B (>80%), and mPanIN-2 (40%) thus confirming previous studies^8^. In contrast, in 3M-old CPK the incidence of mPanIN-1A (100%), mPanIN-1B (100%) and mPanIN-2 (60%) was higher than in age-matched PK mice. Moreover, unlike PK mice, 43% of CPK mice suffered additionally from cystic papillary lesions reminiscent of human IPMN. The mouse lesions, called hereafter as mIPMN, were staged as moderate dysplasias. At the age of 6M the incidence values reached 60% for mPanIN-2, and 40% for moderate mIPMN in CPK. Occurrence and incidence of atypical flat lesions (AFL), described previously for the PK model as well as humans^2^, were similar in both genotypes. As in the PK mice, about 60% of the inspected pancreatic area of CPK was transformed. Exposure of PK and CPK mice to the selective COX-2 inhibitor celebrex at days 1 to 30 postnatally (1M+CX) abolished formation of the described lesions (Fig. 1a). This effect correlated to reduced prostaglandin E_2_ (PGE_2_) levels (see below) indicating that disease progression towards high grade mPanIN and mIPMN lesions is COX-2-dependent.

### Increased COX-2 and PGE_2_ levels in CPK mice

**Figure 2:**
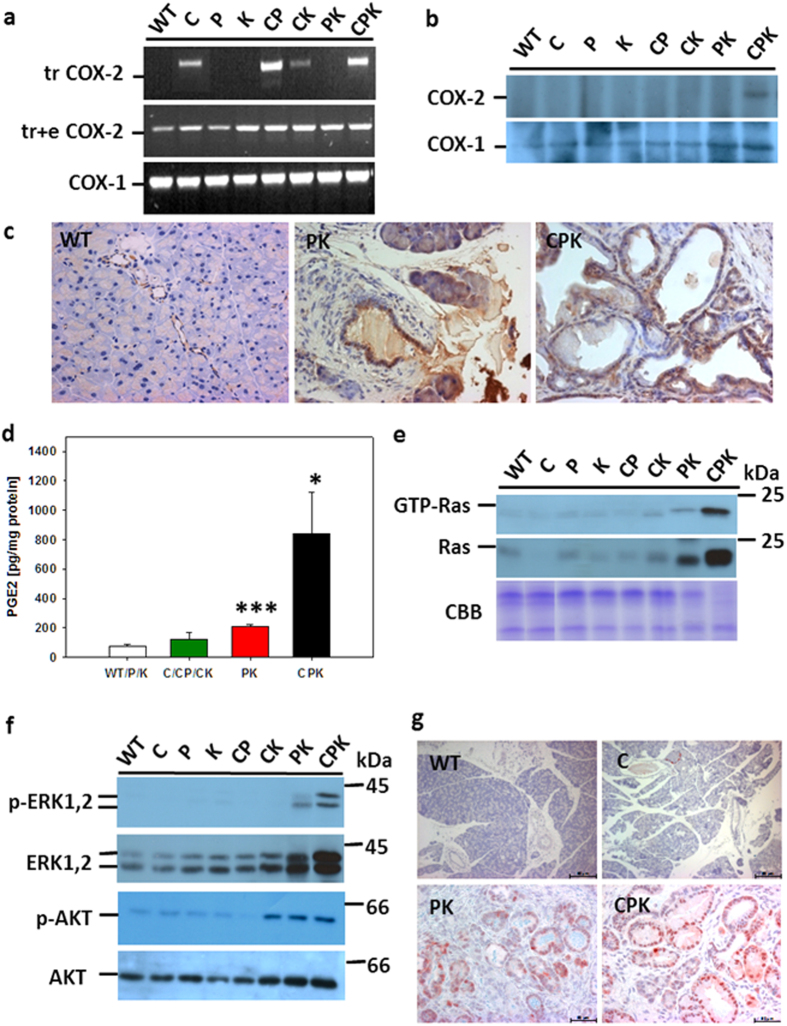
Constitutive COX-2 and Ras activation in PK and CPK mice. **(a)** RT-PCR of transgenic COX-2 mRNA of pancreas from 3-M-old WT, C, P, K, CP, CK, PK, and CPK. Primer sets diagnosed the 3’untranslated region of transgenic (tr) or the transgenic and endogenous (tr+e) COX-2 sequences. As internal control, COX-1 was amplified. **(b)** Immunoblot analysis of COX-2 and COX-1 immunoprecipitated from 1 mg pancreatic protein extract of 3-M-old mice using isozyme-specific antibodies. **(c)** Localization of COX-2 in pancreas of 3-M-old WT, PK, and CPK mice by immunohistochemistry using COX-2-specific antibodies. Note predominant COX-2 expression in duct-like cells and blood vessels in PK and CPK, while only blood vessels were positive in WT pancreas (representative for the WT/P/K group). Magnifications: 40x. **(d)** PGE_2_ level in pancreata of CPK (n = 4) as compared to PK (n = 4), C/CP/CK (n = 2/1/2), and WT/P/K (n = 2/2/2) littermates. Mean values  ±  SEM are given for 1 to 3-M-old mice, *p ≤ 0.05, ***p < 0.001. **(e)** Levels of activated (GTP-Ras) and total Ras in pancreata of CPK and PK mice as compared to littermates of indicated genotypes. GTP-Ras and total Ras were immunodetected using anti-Ras-specific antibodies. CBB-stained protein as loading control served for normalization of activated and total Ras first. Subsequently fold activation of GTP-Ras/Ras was calculated. **(f)** Activation of Ras effector kinases in pancreata of PK and CPK mice. Levels of activated phospho (p)-ERK1,2 and p-AKT as compared to total levels of ERK1,2 and AKT were analyzed by immunoblot using specific antibodies. Expression of phosphorylated forms was computed. **(g)** Immunodetection of p-ERK1,2 in pancreata of WT, C, PK, and CPK mice (3-M-old). Note nuclear signals in the duct-like TC and mPanINs of PK and CPK mice, while the ducts of WT and C mice were negative. Magnifications: 16x (WT, C), 40x (PK, CPK). (**e,f**) are representative for two independent mouse cohorts showing elevated levels of active Ras and p-ERK and p-AKT in PK and CPK mice. The immunohistochemical analysis of p-ERK was performed on separate cohorts of mice (n = 3, each for WT/P/K, C/CP/CK, PK, and CPK). Thus, 5 mice/cohort served to draw the conclusion of activated Ras signaling.

Next, COX-2 expression and PGE_2_ levels in pancreata of CPK as compared to PK mice were checked (Fig. 2a–d). RT-PCR analysis revealed endogenous COX-2 as well as COX-1 expression in all mice independent of the genotype (Fig. 2a). This result reflects the expression of endogenous COX-2 in blood vessels (see below Fig. 2c 15). Using a COX-2 transgene-specific primer set, transgene mRNA expression was verified for all expected genotypes (Fig. 2a). The level of COX isozymes was evaluated by immunoblot analysis of immunoprecipitated COX-2 and COX-1 using isozyme-specific antisera and 1 mg of protein extracted from pancreata of the different genotypes according to published procedures^15^. This analysis showed highest COX-2 protein levels in CPK mice, while COX-1 protein levels were unaffected irrespective of the genotype (Fig. 2b). Subsequent immunostainings revealed COX-2 expression in endothelial cells of pancreata from the WT/P/K group (Fig. 2c) and the COX-2 transgenic mice lines (C/CP/CK, data not shown). Moreover, COX-2 protein was expressed in a pancreatic duct-like compartment in C mice, which reflected stochastic expression in cytokeratin 5-positive cells but also upregulated endogeneous COX-2 in cytokeratin 5-negative (SFig. 4) but cytokeratin 19 (CK19)-positive cells^15^. Lesions of PK and CPK mice were strongly positive for COX-2. Thus, sensitivity of the immunblot analysis was below the COX-2 level in non lesional pancreata of WT/P/K and keratin 5 promoter-driven COX-2 transgenic pancreata of C, CP and CK mice. This result is understandable, given the fact, that duct and endothelial cells represent only about 5–10% of all pancreatic cells resulting in dilution of the COX-2 protein by the bulk of acinar protein. With respect to PK mice, immunohistochemical detection of COX-2 protein in pancreatic lesions confirmed the previous study^8^. Again, levels were found to be below the detection level of immunoblot analysis. While in age-matched pancreata of CPK mice, and as compared to PK mice, COX-2-positive lesions were relatively enriched to a level detectable by the immunoblot approach.

Increased COX-2 protein content in CPK mice correlated to elevated PGE_2_ levels, the predominant PG measured in the pancreas of K5 COX-2 mice^15^. PGE_2_ contents in the transgenic group of C/CP/CK (123.8 pg/mg protein ± 44.2) mice were 1.6-fold higher than in WT/P/K mice (76.9 pg/mg protein  ±  11.6) (p > 0.05). In contrast, lipid levels in PK and CPK mice were elevated 3-fold (p < 0.0001) and 11-fold (p = 0.02), respectively (Fig. 2d). As compared to CPK mice kept on a control diet, PGE_2_ content in pancreata of 1M-old CPK mice exposed to celebrex throughout their postnatal life (30 days) was reduced 2-fold down to 45 pg/mg. Altogether, COX-2 protein expression and activity were higher in CPK than in PK mice.

### Increased Ras activation in CPK mice

Next, levels of activated GTP-bound Ras and total Ras in pancreata of CPK, PK and the other genotypes were compared (Fig. 2e). GTP-Ras as well as total Ras signals were very faint in all lines but PK and CPK mice. The latter displayed high levels of total as well as activated Ras. Semiquantitative evaluation of signal intensities, after normalization to total CBB-stained protein and total Ras^15^, revealed a 3-fold increase in CPK as compared to PK mice. However, the levels in PK pancreata were about 1.2-fold and 1.4-fold higher than in C/CP/CK and WT/P/K samples, respectively. In line, immunoblot detection of Ras downstream effector protein kinases ERK and AKT in non-phosphorylated (inactive) as compared to phosphorylated (p, active) forms showed about 2-fold higher levels of p-ERK-1,2 in CPK as compared to PK. Change was about 16-fold in CPK and 9-fold in PK as compared to the two other groups (Fig. 2f). With respect to pAKT, CPK and PK exhibited similar levels that were about 2.5-fold higher than in C/CP/CK and about 4-fold higher than in WT/P/K. To identify the cellular pancreatic compartment exhibiting activated Ras signaling, immunostaining of p-ERK-1,2 was done (Fig. 2g). While the activated kinase was not observed in WT/P/K or C/CP/CK pancreata, signals were found predominantly in the nuclei of TC and mPanIN in both PK and CPK specimens. Elevated levels of total and activated Ras were accompanied by increased levels of total and activated downstream effector kinases, thus probably indicating an expansion of proliferating ductal cells.

### Increased proliferation index in the ductal compartment of CPK mice

Proliferation, measured by quantifying Ki67-positive nuclei in the pancreatic ductal compartment (SFig. 5), revealed indices of 0.55% (4/767 cells) in WT/P/K, 1.37% (25/1473 cells) in C/CP/CK transgenic (p > 0.05), 10.9% (213/1944 cells) in PK mice (p < 0.0001), and 22.1% (528/2627 cells) in CPK mice (p = 0.002), i.e. a two-fold proliferation increase in CPK versus PK mice (p < 0.05).

### COX-2-dependent transcriptome signature in early-stage lesions: Upregulation of Notch signaling

**Figure 3:**
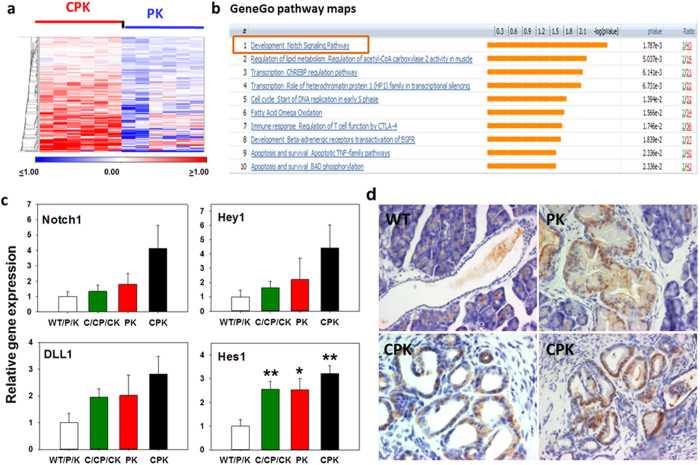
Differential transcriptomes of laser capture microdissected mPanIN-like lesions of PK and CPK mice: Validation of differentially expressed Notch1 signaling in CPK. **(a)** *In vivo* expression profile of laser capture microdissected from mPanIN-like lesions of PK and CPK mice (n = 6, each). The heat map represents genes from 2 class t-test p ≤ 0.005 (without multiple testing correction), 191 transcripts with significant upregulation in CPK vs PK were selected. Each row represents log_2_ expression ratios of an individual gene and each column indicates an individual sample. Expression ratios are colored according to the scale bar: blue >2-fold downregulation, red >2-fold upregulation. **(b)** Top 10 functional processes affected by COX-2 overexpression in the PK genetic background. Data analysis among upregulated genes from the 3 months-old CPK and PK gene expression sets resulted in significant enrichment for gene ontology processes related to Notch signaling in development, regulation of lipid metabolism, immune response, cell cycle, survival and apoptosis. **(c)** Relative gene expression levels of Notch1, Hey1, DLL1, and Hes1 in pancreata of control WT/P/K mice (n = 3) compared to C/CP/CK (n = 3), PK (n = 5) and CPK (n = 5) mice as measured by qRT-PCR. COX-1 amplicons served for normalization. Data is given as mean  ±  SEM. Statistical significance (*p ≤ 0.05, **p ≤ 0.01) was evaluated by Student’s t-test. **(d)** Expression of Hes1 in pancreata of WT, PK, and CPK mice as detected by immunohistochemistry using anti-Hes-1 antibodies. Note unspecific signals in WT tissue, while nuclear Hes1 signals were observed in TC and mPanIN-like lesions of PK and CPK specimens. Nuclei are stained with hematoxylin. Magnifications: 40x (WT), 63x (PK, CPK).

To evaluate the COX-2-dependent transcriptomic signature of early-stage lesions, PK and CPK pancreatic specimens (n = 6 individual samples, each) were subjected to laser microdissection (SFig. 6) and subsequent genome wide expression profiling using mouse IIlumina mouse sentrix-8 chips. Two class t-test was performed to find genes significantly differentially expressed in CPK vs PK samples (Fig. 3a). At p < 0.05 (without multiple testing correction) 3872 transcripts were found to be significantly regulated in CPK samples. Pathway enrichment analysis, performed to explore biological context of the selected genes using the Kyoto Encylopedia of Genes and Genomes (KEGG) database tool, revealed downregulated gene sets which are relevant in apoptosis, p53 signaling, adherens junctions and regulation of the actin cytoskeleton (STable 2). Differentially elevated transcripts in CPK lesions related to signaling of cell cycle, mismatch repair, and MAPK signaling were identified. Furthermore, transcripts known to be elevated in cancer including pancreatic cancer were found. Since no genes were found at p < 0.05 with multiple testing correction, best genes were filtered at p < 0.005 and subsequently carefully evaluated. 191 transcripts (p ≤ 0.005) were found to be significantly upregulated in CPK samples. Analysis of this set of candidates by KEGG but also MetaCore’s GeneGo platform revealed developmental Notch signaling pathway ranking highest among the pathway maps (p < 0.005) followed by pathways involved in lipid metabolism, immune responses, cell cycle, survival, and apoptosis as well as protein processing in endoplasmic reticulum, peroxisomes, and p53 signaling (Fig. 3b, STable 3).

To validate upregulation of Notch signaling components in CPK, qRT-PCR was performed on total RNA isolated from independent pancreatic samples (Fig. 3c). As compared to WT/P/K samples, CPK pancreata showed up with highest regulation in the components checked namely Notch1, DLL1, Hey1 and Hes1. Additional immunostaining of Hes1 protein revealed nuclear signals in ductal lesions of PK and CPK mutants, while the duct compartment of WT/P/K mice was negative (Fig. 3d). Additionally, a primary CK5–positive cell culture established from C-transgenic pancreatic ductal cysts coexpressed COX-2 and Hes1 along with CK19 and carbonic anhydrase II (CA-II) but not elastase (SFig. 7).

**Figure 4:**
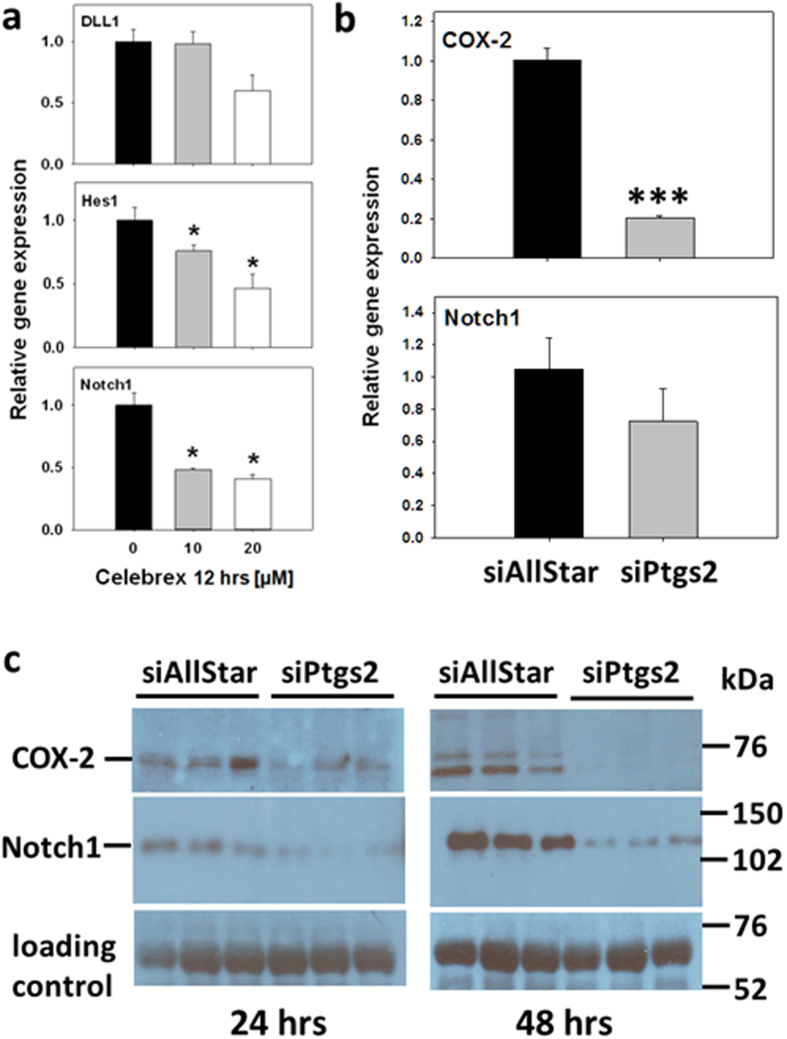
COX-2-dependent modulation of Notch1 expression. **(a)** Relative gene expression levels of DLL1, Hes1 and Notch1 in Capan-1 cells as measured by qRT-PCR. Capan1 were treated 24 hours after seeding with ethanol as control (0) or with 10 or 20 μM celebrex for 12 hours. Amplification of COX-1 was performed for normalization. Data is presented as mean  ±  SEM of n = 3 cultures, each with 2 technical replicates each. Student’s t-test was performed to analyze for statistical significance (*p < 0.05). **(b)** Notch1 mRNA expression in BxPC3 cells after siRNA-mediated Ptgs2 (COX-2) knockdown. BxPC3 cells were transfected with 5 nM of siPtgs2 or 25 nM siAllStar negative control. Plates were incubated, and Ptgs2 was monitored along with Notch1 expression at 24 hours by qRT-PCR. Data is presented as mean  ±  SEM of n = 3 cultures, each with 2 technical replicates each. (Student’s t-test: ***p < 0,001). **(c)** Reduced Notch1 protein levels in BxPC3 cells after siRNA-mediated Ptgs2 (COX-2) knockdown. BxPC3 cells were transfected with 5 nM of siPtgs2 or 25 nM siAllStar negative control and incubated for 24 or 48 hours. COX-2 protein was monitored along with Notch1 protein by immunoblot analysis. Data is presented as mean  ±  SEM of n = 3 cultures, each. Semiquantitative evaluation revealed 1 ± 0.227 arbitrary units for COX-2 in siAllStar negative control and 0.228 ± 0.018 in siPtgs2 group indicating an about 4-fold knockdown of COX-2 protein at 48 hours. This effect was only 1.4-fold at 24 hours as calculated from siAllStar negative control (1 ± 0.149) versus siPtgs2 (0.725 ± 0.211) samples.

Dependency of Notch1, Hes1 and DLL1 mRNA expression levels on COX-2 activity was further substantiated *in vitro* in the pancreatic cancer cell line Capan-1, a K-Ras mutant cell line^21^. In cultures treated with increasing concentrations of celebrex to inhibit COX-2 activity, relative gene expression of Notch1 and Hes1 as well as DLL1 was reduced as compared to vehicle treated cells (Fig. 4a). Since Notch1 has been shown to be a downstream target of oncogenic H-Ras^22^, we performed an additional siRNA-mediated knockdown of Ptgs2 transcripts to address if Notch1 is under regulation of COX-2. Therefore, BxPC3 pancreatic carcinoma cells which are known to be wild-type in K-Ras^21^ were used. As a consequence of Ptgs2 mRNA and protein knockdown (Fig. 4b,c), steady-state levels of Notch1 receptor mRNA and protein were downregulated too (Fig. 4b,c). Taken together, the result indicates that Notch1 is under regulation of COX-2, even in the absence of oncogenic K-Ras.

### Notch expression in human IPMN

**Figure 5:**
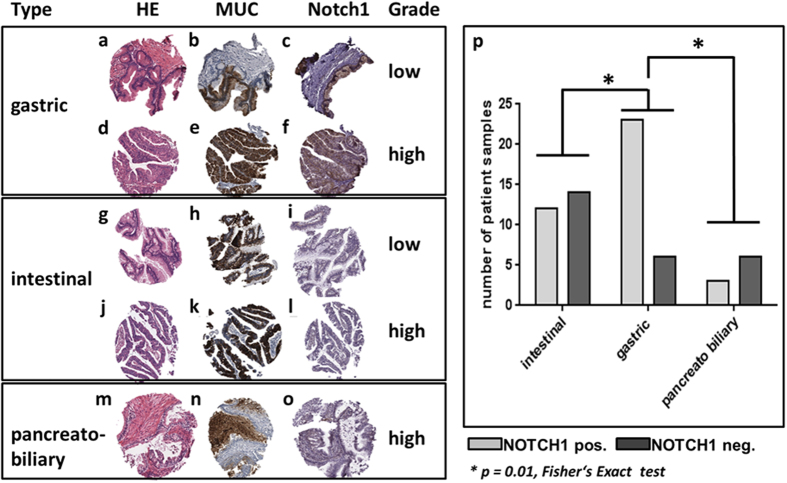
Notch1 expression in human IPMN. Representative microphotographs of gastric, intestinal and pancreatobiliary IPMN stained HE (**a,d,g,j,m**) and immunostained for corresponding expression of MUC5AC (**b,e**), MUC2 (**h,k**), MUC1 (**n**) and Notch1 (**c,f,i,l,o**). Low **(a–c)** and high-grade **(d–f)** gastric IPMN with typical MUC5AC expression **(b,e)** and positivity for Notch1 **(c,f)**. Low **(g–i)** and high-grade **(j–l)** intestinal IPMN with typical MUC2 expression **(h,k)**. Notch 1 is weakly expressed in the low-grade IPMN **(i)**, whereas no positivity is detected in the high-grade lesion **(l)**. High-grade **(m–o)** pancreatobiliary IPMN with typical MUC1 expression **(n)** and weak positivity for Notch 1 **(o)**. (**p**) Statistical analysis by Fischer’s Exact Test revealed significant higher Notch1 positivity in the gastric type IPMN as compared to intestinal and pancreatobiliary types of IPMN.

To underscore the significance of our experimental animal studies, deciphering unique formation of IPMN in the CPK as compared to PK mice, human IPMN tumor samples were comprehensively examined by tissue microarray for Notch1 (Fig. 5). A set of 64 low (mild to moderate dysplasia) to high-grade (carcinoma *in situ*) IPMN lesions, known to express COX-2^15^, were evaluated. Overall, 64% of all IPMN expressed Notch1. Further stratification of IPMN lesions (according to expression profile of Muc1, Muc2, and Muc5) into intestinal, gastric and pancreatobiliary types^23^ revealed Notch incidences of 46%, 79%, and 33%, respectively. The frequency of Notch1 positivity was significantly higher in gastric-type IPMN than in other subtypes (Fig. 5p), but no significant association with the degree of dysplasia was found. This finding suggests that Notch1 is particularly associated with early-stage progression of the gastric type IPMN.

## Discussion

In this study it is demonstrated that the extent of overall transformation in the pancreas, i.e. the replacement of acinar tissue by tubular complexes, PanIN, IPMN, atypical flat lesions (AFL) and inflammatory infiltrates is similar in PK and CPK mouse lines. However, concomitant expression and elevated activities of COX-2 and K-Ras^G12D^ in the CPK pancreas accelerated appearance of CK19-positive early stage PanIN and cystic IPMN lesions. Recently, AFL found in the PK line were characterized by a high proliferation index and cytological duct atypia. AFL were also found with 100% prevalence in CPK mice with increasing age. Molecular and immunohistochemical analysis indicated that AFL might also be precursor lesions of PDAC resembling the situation in patients with familial PDAC^2^.

In an 18 months follow up of cohorts no carcinomas were found in PK and CPK genotypes. Thus, the accelerating effect of COX-2 seems to be restricted to the early preinvasive stages. This view is in line with the observation that either a loss of Smad4^24^ or an elastase promoter-driven overexpression of transforming growth factor α (TGFα)^25^ in the PK model are essential for development of lesions similar to IPMN. Both, PK and CPK developed early on tubular complexes at a comparably high incidence allowing the hypothesis that these lesions could be precursors of both PanIN and IPMN. Importantly, at the age of 6 months, around 40% of CPK mice displayed cystic lesions classified as alcian blue/PAS-positive IPMN with moderate dysplasia, while PK were devoid of this type.

Celebrex inhibited in PK and CPK mice the pathological pancreatic transformation along with a suppression of PGE_2_ levels allowing the conclusion that the observed phenotypic changes were COX-2-dependent. This interpretation is supported by a report, whereby the COX-2-selective inhibitor nimesulfide delays the progression of PanIN in the PK mutants^19^. COX-2 has been shown to be key to inflammation-driven K-Ras^G12D^ initiated pancreas cancer^8^,^13^,^14^. Most convincingly, mice with simultaneous expression of COX-2 and oncogenic Ras^G12D^ in the acinar compartment developed severe chronic pancreatitis characterized by fibrosis, inflammation, loss of acinar cells and PanIN formation^14^. It was shown that inflammatory stimuli induced greater and more prolonged Ras signaling, NF-kB activity and COX-2 in the context of oncogenic K-Ras. Different from our study, synergistic effects of COX-2 and oncogenic K-Ras in promotion of severe chronic pancreatitis and PanIN formation resulted from co-overexpression of both genes in one and the same cellular compartment^14^. In this study, accelerated formation of preinvasive lesions in the acinar K-Ras^G12D^ background is triggered from stochastic COX-2 overexpression, as driven by cytokeratin 5 promoter in the duct compartment, perpetuating the upregulation of endogenous COX-2 in the duct compartment itself and possibly in K-Ras^G12D^-driven lesional duct structures. Moreover, COX-2-derived prostaglandins obviously activate Ras signaling in a paracrine manner, since phospho-ERK is more strongly upregulated in lesional pancreas of CPK mice as compared to PK mice.

Total Ras was found to be increased in PK and CPK mice, although the Ras^G12D^ knockin per se implies that after Cre-mediated recombination the gene is transcribed with a “natural” expression rate under control of the endogenous native K-Ras promoter in centroacinar as well as acinar cells in the adult. In previous reports by Hingorani *et al*.^8^ and Huang *et al*.^26^, 2-month-old PK mice did not show elevated levels of total Ras as compared to controls. According to published data using colon cells^27^ and our own data with BxPC3 cells (SFig. 8), COX-2-derived prostaglandins obviously modulate the Ras-MAPK signaling but neither upregulate levels of total Ras nor total ERK or AKT. In our *in vivo* study, 3-months-old mice exhibiting already early-stage pancreatic lesions to a great extent were analyzed. One explanation for increased steady-state levels of total Ras could be the increased transcriptional activity on the Ras-loci in early-stage lesions. Ras gene amplification also cannot be excluded.

In contrast, a body of evidence indicates that COX-2-dependent signals activate Ras on a non-mutational level. The COX-2-selective inhibitor celebrex normalized elevated GTP-Ras levels in K5 COX-2 mice^15^ despite wild-type K-Ras sequences in pancreatic cancer-relevant codons 12, 13, and 61 (SFig. 1) as well as in BxPC3 cells *in vitro* (SFig. 8) and in accordance, relative GTP-Ras levels as well as phosphorylated ERK-1,2 were higher in pancreatic ducts of early stage lesions of the CPK mutants than in the PK mutant. PG synthesized via COX-2 bind to and activate specific G-protein-coupled seven transmembrane receptors located on the plasma membrane^10^. These will in turn activate effector molecules upstream of Ras, whereby a PG-stimulated transactivation of a growth factor receptor tyrosine kinase such as EGFR (by ligands such as TGFα and amphiregulin) or HER activation alone is a possible scenario in this process^28^,^29^,^30^,^31^,^32^.

Activated Ras initiates downstream signaling pathways such as the MAPK and PI3K/AKT tracks among many others^7^,^33^, leading to transcriptional activation of target genes including amphiregulin and COX-2, thus promoting growth and survival. Indeed, constitutive COX-2 overexpression is a consequence following oncogenic K-Ras mutation as observed here and by others^8^,^28^,^29^,^30^. Taken together, double positive feed forward loops between Ras and COX-2 is proposed for pancreatic lesional cells with the consequence of permanently amplified Ras signaling in CPK mice as compared to PK mice. Beyond proinflammatory mediators such as COX-2-derived prostaglandins, cytokines and growth factors that are probably activated by Ras, and that may also lead to further Ras activation, have yet to be defined in more detail for the IPMN-PDAC sequence as has been done for the PanIN-PDAC sequence^7^.

Notch signaling is fundamental to pancreas development and regulates cell fate decisions and differentiation routes^34^. In adult healthy pancreas, differential expression of Notch receptors has been reported for the individual cellular compartments of pancreas^35^ including terminal ductal epithelium and centroacinar cells^36^, discussed to be a progenitor pool in adult pancreas^37^. Moreover, Notch1 function is required for exocrine regeneration after acute pancreatitis^38^. In human PanIN-PDAC sequence, profiling of Notch signaling components revealed activation of a Notch signaling module with predominant activation of Notch2, Notch3, Notch4, Dll1, and Jag1 as well as target genes Hes1, Hes4, Hey1, and HeyL^36^. This pattern of overexpression argued for protumorigenic effects. In line with expression data, Notch2 rather than Notch1 seems to be required for progression of mPanIN and PDAC, at least in the background of a P48Cre/K-Ras^G12D^ mutant line^8^,^39^. Moreover, genetic inhibition of Notch signaling resulted in a transient delay in mPanIN formation in the P48Cre/K-Ras^G12D^ mutant^40^. However, the protumorigenic role of Notch signaling^41^ is still debated, since genetic knockout of Notch1 in Pdx-1Cre/K-Ras^G12D^ mutants increased tumor incidence and accelerated tumor progression arguing for a tumor suppressive effect^42^.

Recently, a role of Notch1 signaling has been implicated in PanIN/IPMN formation in P48-Cre/K-Ras^G12D^ mouse mutants with elastase promoter-driven overexpression of TGFα. These mice developed IPMN lesions^25^ as in the CPK mice. In our study, an unbiased transcriptomic microarray analysis with bioinformatic data integration and subsequent validation by qRT-PCR unraveled Notch signaling components (Notch1, Hes1) to be differentially upregulated in early-stage, laser microdissected lesions in CPK mutants as compared to the PK mutants. Hes1 protein was expressed in the nuclei of pancreata indicating active Notch1 signaling, a result that correlated with exclusive outgrowth of IPMN-like lesions in the CPK line. *In vitro* studies, applying a COX-2-selective inhibitor to COX-2-positive pancreatic cancer cells, revealed that expression of Notch1, Hes1 and DLL1 depended on COX-2-mediated prostaglandin synthesis. Furthermore, the positive regulation of Notch1 level by COX-2 was evident from a siRNA-mediated Ptgs2 (COX-2) knockdown experiment in a pancreas cancer cell line BxPC3 harboring a wild-type K-Ras. In the context of gastric cancer progression, activated Notch1 signaling occurred through COX-2. Here, direct targeting of the COX-2-promoter by the Notch1 receptor intracellular domain as well as C promoter-binding factor 1 was found^43^. Taking these studies together, a positive feedback loop between Notch1 and COX-2 is postulated. Of course, it needs future experiments to clarify if this regulation occurs directly via COX-2-mediated prostaglandins and/or indirectly via sustained activated/oncogenic Ras, in view of the facts that COX-2 inhibition by celebrex also inhibited Ras activation in BxPC3 cells *in vitro* and that Notch1 signaling is also among key downstream effectors of oncogenic Ras in different cellular contexts^22^,^44^,^45^,^46^.

Our experimental animal study with a P48Cre/K-Ras^G12D^.K5-COX-2 compound model revealed a yet unknown link between protumorigenic COX-2 and Notch1 and activated K-Ras driving mIPMN onset. The relevance of this data is substantiated by the finding that human COX-2-positive IPMN specimens^23^ were positive for Notch1 expression, with a high prevalence in the gastric-type IPMN.

Taken together, our data suggest a crucial role of the COX-2-Notch1 axis cooperating with oncogenic K-Ras to promote not only the formation of mPanIN^47^ but also formation of neoplastic cystic IPMN.

## Methods

### Mouse strains and tumor models

NMRI.K5 COX-2 transgenic line 675^+/+^ (https://mito.dkfz.de/mito/Tumor%20model/10455) was kept as described^48^,^49^. This line and the P48-Cre/K-Ras^G12D/wt^ mouse line^8^ were backcrossed onto the C57BL6/N background (Charles River, MA) for 10 generations to generate heterozygous C57BL6/N.K5 COX-2/675^+/wt^ and C57BL6/N.P48-Cre/K-Ras^G12D/wt^ mice, respectively. Breeding of C57BL6/N compound mutant mice resulted in F1 with the following genotypes: K5 COX-2/675^+/wt^.P48-Cre/K-Ras^G12D/wt^ (CPK), P48-Cre/K-Ras^G12D/wt^ (PK), K5 COX-2/675^+/wt^ (C), wild type (WT), P48-Cre (P), K-Ras^G12D/wt^ (K), K5 COX-2/675^+/wt^.P48-Cre (CP), and K5 COX-2/675^+/wt^.K-Ras^G12D/wt^ (CK). Offsprings were treated with celebrex-containing Kliba 3437 diet (1500 ppm) starting 1 day after birth through weaning mothers for 4 weeks. Necropsies were snap-frozen in liquid N_2_ or processed for histological stainings. All animal breedings and animal care as well as all animal experiments were performed according to the national guidelines and were approved first by an institutional review board/ethics committee headed by the local animal welfare officer (Dr. Michaela Socher) of the German Cancer Research Center Heidelberg, Germany. All experiments were in addition approved by the responsible national authority, the local Governmental Committee for Animal Experimentation (Regierungspräsidium Karlsruhe, Germany) under licenses G053/00 and G36/08 and were carried out accordingly.

### Cell culture experiments

Human pancreatic Capan-1 and BxPC3 cells, generously provided by O. Ströbel from Department of Surgery at University of Heidelberg, were cultured in DMEM 4.5g/l glucose (PAA, Pasching/Austria), 10% FBS (Biochrom, Berlin/Germany), and 1% Pen/Strep (Life Technologies, Darmstadt/Germany) at 37 °C and 5% CO_2_. Twenty four hours after seeding of Capan-1 (10.000/cm^2^), cells were exposed to increasing concentrations of celebrex or ethanol as vehicle for 12 hours.

### Ptgs2(COX-2) knockdown *in vitro*

Shortly prior to transfection, 2 × 10^5^ BxPC3 cells in 1100 μl RPMI medium (ATCC, VA) were seeded per well of a 24-well plate and incubated under conditions described above. Into serum-free culture medium, siRNA (S100301525) from the FlexiTube Gene Solution GS5743 for Ptgs2 kit (QIAGEN, Hilden/German) were diluted to a final concentration of 5 nM. To the diluted siRNA, 6 μl of HiPerFect Transfection Reagent (QIAGEN) was added and mixed by vortexing. As a negative control, a final concentration of 25 nM AllStars Negative Control siRNA (QIAGEN) was used. Transfection complexes were incubated 10 minutes at room temperature and added dropwise onto the cells while swirling. Plates were incubated, and Ptgs2 knockdown was monitored along with Notch1 expression at 24 and 48 hours (n = 3 each).

### Human biopsy specimens and immunohistochemistry

The human tissues were retrieved from the archives of the Institute of Pathology of the University of Heidelberg, Germany and of the Technische Universität München, Germany, and used in a fully anonymized way in accordance with local ethical regulation (Heidelberg: 301/2001 and Munich: 1967/2007) and the Declaration of Helsinki. Written informed consent regarding tissue collection and analysis for research purposes was obtained from all patients included in the study. Formalin-fixed paraffin-embedded samples of resected IPMN were used to construct tissue microarrays (TMA) using a manual tissue arrayer (Beecher Instruments, WI), as previously described^50^. IPMN were classified according to the histological subtype (gastric, intestinal, pancreatobiliary and oncocytic) and the degree of dysplasia (low-grade, high-grade and IPMN with associated invasive adenocarcinoma). The histological subtype was determined by morphology and the immunohistochemical expression profile of MUC1, MUC2, and MUC5AC, according to WHO criteria (Bosman FT, Carneiro F, Hruban RH, Theise ND (Eds.): WHO Classification of Tumours of The Digestive System. IARC: Lyon 2010). Altogether, 28 intestinal (15 low-grade, 6 high-grade, 7 with associated invasive colloid adenocarcinoma), 30 gastric (27 low-grade, 3 high-grade) and 9 pancreatobiliary IPMN (1 low-grade, 8 high-grade) were included. Immunohistochemistry was performed using an automated system (DiscoveryXT, Roche, Mannheim/Germany) with modified protocols for human tissues. Briefly, tissue sections were deparaffinized, rehydrated, and subjected to peroxidase block and antigen retrieval, if required. To block unspecific antibody binding, blocking serum (KPL Kirkegaard and Perry Laboratories, MD) was applied for 30 minutes. Isotype-matched IgG served as negative controls. The following primary antibodies were used: anti-MUC1 (clone Ma695, Novocastra/Menarini Diagnostics, Berlin/Germany. 1:100), anti-MUC2 (clone CCP558, Novocastra/Menarini Diagnostics. 1:100), anti-MUC5AC (clone CLH2, Chemicon, CA. 1:1000), and anti-Notch1 (clone D1E11, Cell Signaling, MA. 1:200). Results of the immunohistochemical analysis were compared using the Fisher’s Exact test for comparison of categorical parameters. Significance was defined as p ≤ 0.05.

### Immunohistochemistry and immunofluorescense of mouse tissue

Five μm cryosections of mouse pancreas were used for hematoxylin/eosin (HE) stainings. Histopathologic analysis and grading of lesions was done by I. Esposito if not stated otherwise according to published consensus^3^. Cryosections were fixed in acetone for 10 minutes (min), washed in PBS, blocked in 1% BSA in PBS for 1 hour (h) and incubated with anti-CK19 primary antibody (1:50), kindly provided by R. Kemmler (Freiburg/Germany), polyclonal rabbit anti-keratin 5 (Convance, Berkeley, CA) or anti-Ki67 #Sp6 (1:100) (Thermo Scientific, CA) overnight at 4 °C. Appropriate fluorochrome-coupled secondary antibodies (1:200) were applied for 1 h at room temperature (RT). Hoechst dye was used to stain nuclei. Sections were mounted in Dako mounting medium (Dako, Hamburg/Germany). The proliferation index is given as the percentage of Ki67-positive duct cells. The number of Ki67-positive ductal cells in a total of randomly selected 20 microscopic fields per section of individual mice (n = 3–5) was counted along with the number of all nuclei in the ductal compartment within the chosen fields. COX-2, Hes1, and p-ERK1, 2 were detected in deparaffinized tissue specimens after antigen retrieval (20 min at 50 °C in 10 mM sodium citrate pH 6.0), block of endogenous peroxidases in 3% H_2_O_2_ in methanol for 10 min, and block in 1% BSA in PBS for 1 h at RT, and incubation in a 1:50 dilution of anti-COX-2 (Santa Cruz, CA), anti-Hes1 (Toray Industries, Tokyo/Japan) or anti-p-ERK1,2 (Cell Signaling) overnight at 4 °C. Slides, incubated with appropriate peroxidase-coupled secondary antibody (1:200) for 1 h at RT, were incubated with HRP substrate solution (DAB/H_2_O_2_, Sigma, Munich/Germany). Nuclei were counterstained with hematoxylin for 1 min and mounted with Eukitt (R. Langenbrinck, Teningen/Germany). Sections incubated without primary antibody were included as negative controls. Microscopy was performed using Axioskop 2 microscope and Axiovision software (Carl Zeiss, Gottingen/Germany).

### Ras activation assay

GTP-Ras was measured using the Ras activation assay kit (Upstate, NY) following the manufacturer’s instructions and as described^15^. Briefly, 2 mg of pancreatic protein served to pull down GTP-Ras (activated Ras). Affinity precipitated GTP-Ras or 100 μg of pancreatic protein were separated in 15% gels by SDS-PAGE. GTP-Ras and total Ras were immunodetected using anti-Ras-specific antibodies. As a loading control, 80 μg of protein of each sample were separated by SDS-PAGE, and stained with coomassie brilliant blue (CBB).

### Immunoblot

Immunoprecipitation of COX isozymes and immunoblot analysis were performed as described using isozyme specific antisera^48^,^49^. For immunodetection of ERK and AKT proteins, frozen pancreas powder was homogenized in RIPA buffer (150 mM NaCl, 20 mM Tris/HCl pH 8.0, 1% Triton X-100, 1% C24H39NaO4, 1 mM PMSF, 2 mM EDTA, 0.1% SDS, 25 mM NaF, 1 mM Na_3_VO_4_, 10 μg/ml aprotinin, 10 μg/ml leupeptin, 0.2 mg/ml α_2_-macroglobulin). Immunoblots were incubated with anti-AKT, anti-p-AKT, anti-ERK1,2, anti-p-ERK1,2 antibodies (Cell Signaling, 1:1000 dilution) or anti-Notch1 antibodies (Cell Signaling; 1:2000) overnight at 4 °C. Peroxidase-coupled secondary antibodies (1:8000- 1:10000 diluted) were incubated at RT for 1 h. For detection of signals, the ECL western blotting detection kit was used (Amersham BioSciences, Freiburg/Germany). For Notch1 blots, the TE671 cell lysate served as positive control (Santa Cruz Biotechnology, Heidelberg/Germany, SC-2416). Cell monolayers transfected for 24 and 48 hours with siPtgs2 or siAllstars negative (n = 3, each) were washed twice with PBS and frozen at −20 °C prior to extraction with HP-buffer. The extracted protein served, after precipitation with ethanol and denaturation in Laemmli sample buffer (LSB), as a loading control. The cell monolayers were then reextracted with 2x LSB and denatured for immunodetection of Notch1 and COX-2.

### Determination of PGE_2_ levels

PGE_2_ contents (mean pg/mg protein ± SEM) were determined as described^49^. By means of a PGE_2_-specific enzyme immunoassay, lipid levels were quantified and normalized to the amount of extracted protein.

### Expression profiling

Pulverized pancreatic powder generated under conditions of liquid N_2_ by means of a ball mill (Braun, Melsungen/Germany) served to extract total RNA using the RNeasy mini kit (QIAGEN). Total RNA of cells was isolated using TRIzol (Life Technologies). RNA integrity was determined using RNA 6000 Nano Lab on Chip kits and Agilent 2100 Bioanalyzer (Agilent, CA). RNA concentrations were determined by Nano Drop (Peqlab, Erlangen/Germany). After cDNA synthesis using the GeneAmp RNA-PCR core kit (Applied Biosystems, CA), PCR or quantitative PCR (qRT-PCR) were performed using respective forward and reverse primers (STable 1). Plates were analyzed by a 7900HT Fast Real-Time PCR System (Applied Biosystems) according to the manufacturer’s protocol.

### Laser capture microdissection (LCM)

Ten μm sections, placed onto PALM membrane slides (Carl Zeiss), were stained with hematoxylin prior to LCM with a PALM MicroBeam system (PALM Microlaser Technologies, Munich/Germany). mPanIN-like lesions and tubular complexes (TC) of 3 months-old PK, and CPK pancreatic specimens were dissected individually by catapulting the material into the PALM adhesive caps (SFig. 6).

### RNA extraction from LCM tissue

Total RNA was isolated with RNeasy micro kit (QIAGEN) following manufacturer’s instructions. RNA integrity number (RIN) was measured by Total RNA 6000 Pico Nano Lab on Chip kits and Agilent 2100 Bioanalyzer (Agilent). Samples with RIN ≥7 were utilized.

### *In vitro* amplification and transcription

The WT-Ovation^TM^ Pico RNA amplification system (Nugene, Saffron Walden/UK) was used to amplify cRNA that was synthesized from 500 ng of total RNA keeping with the appended protocols.

### Microarray hybridization of LCM samples

cRNA was labeled with biotin by the Encore BiotinIL Module (Nugene). For hybridization onto IIlumina mouse sentrix-8 chips (Illumina Inc, CA), biotin-labeled cRNA samples were prepared according to Illumina’s recommended sample labeling procedure based on the modified Eberwine protocol^51^. Hybridization was performed at 55.4 °C in GEX-HCB buffer (Illumina) at a concentration of 100 ng cDNA/μl and kept unsealed in a wet chamber for 20 h. Slides were scanned with Beadstation array scanner.

### Bioinformatic analysis

Generation of expression matrices, data annotation, filtering and processing were done using our in-house TableButler software package (http://angiogenesis.dkfz.de/oncoexpress/software/tablebutler/). All microarray statistics were done using the in-house SUMO software package (http://angiogenesis.dkfz.de/oncoexpress/software/sumo/). Data analysis was done by normalization of the signals using quantile normalization algorithm without background subtraction. Arithmetic average intensities across all samples for each gene were computed. Extremely low abundant genes (average intensities <100) were excluded from the analysis leaving 26,436 transcripts. Ratios were computed by dividing each individual gene by the median average for each individual gene and Log_2_ transformed (virtual pool normalization). A correlation map (linear regression) was generated to demonstrate the similarity within and difference between the animal groups. Two class t-test was performed to filter significantly differentially regulated genes. The obtained gene expression profiles were visualized as heatmaps. MetaCore’s GeneGo (Thomson Reuters, NY, http://genego.com) software tool as well as the Kyoto Encylopedia of Genes and Genomes (http://genome.jp/kegg) database were applied to appraise biological pathways involving our candidate genes in the two sample sets (PK vs CPK). Microarray data were submitted to and are available online at ArrayExpress http://www.ebi.ac.uk/arrayexpress under the accession no. E-MTAB-2688.

## Additional Information

**How to cite this article**: Chiblak, S. *et al*. K-Ras and cyclooxygenase-2 coactivation augments intraductal papillary mucinous neoplasm and Notch1 mimicking human pancreas lesions. *Sci. Rep.* **6**, 29455; doi: 10.1038/srep29455 (2016).

## Supplementary Material

Supplementary Information
