# Supplementary material for: K-Ras and cyclooxygenase-2 coactivation augments intraductal papillary mucinous neoplasm and Notch1 mimicking human pancreas lesions
Source: Sci Rep. 2016 Jul 6;6:29455. doi: 10.1038/srep29455 (PMC4933934; doi:10.1038/srep29455)
Supplement: Supplementary Information [file srep29455-s1.pdf]

K-Ras and cyclooxygenase-2 coactivation augments intraductal papillary mucinous neoplasm and Notch1 mimicking human pancreas lesions

Sara Chiblak[1,2], Brigitte Steinbauer[1], Andrea Pohl-Arnold[1], Dagmar Kucher[1], Amir Abdollahi[2], Christian Schwager[2], Birgit Höft[3], Irene Esposito[4,§], Karin Müller-Decker[1,§,*]

[1]*Tumor Models, Deutsches Krebsforschungszentrum (DKFZ),69120 Heidelberg, Germany*
[2]*German Cancer Consortium (DKTK); Molecular & Translational Radiation Oncology, Heidelberg Ion Therapy Center (HIT), Heidelberg Institute of Radiation Oncology (HIRO), University of Heidelberg Medical School and National Center for Tumor Diseases (NCT), DKFZ, 69120 Heidelberg, Germany*
[3]*Birgit Höft, Division of Cancer Epidemiology, DKFZ, 69120 Heidelberg, Germany*
[4]*Institute of Pathology, Heinrich-Heine-University of Duesseldorf, 40225 Duesseldorf, Germany*
[§]*shared senior authorship*

***Corresponding author:**
K. Müller-Decker
Deutsches Krebsforschungszentrum Heidelberg,
Im Neuenheimer Feld 280, 69120 Heidelberg,
Phone: 49-6221-424 531
Fax: 49-6221-424 406
E-mail: K.Mueller-Decker@dkfz.de

Supplementary Information

Materials and Methods:

Extraction of genomic DNA. For genotyping of genetically manipulated mouse lines, DNA was extracted from tail biopsies as described previously [1]. 100 ng DNA was amplified using specific forward and reverse primers mentioned (STable 1). For genomic DNA extraction from pancreas, 80 mg of tissue powder were processed by the QIAGEN midi kit (QIAGEN). Measurement of optical density at 260 and 280 nm (Nano Drop) served to determine DNA concentrations.

PCR and pyrosequencing. For pyrosequencing of K-Ras cancer-relevant sequences, amplification for codons 12/13 and 61 with the least surrounding genetic material was carried out by a PCR using 50 ng of pancreatic DNA in a 50 µl reaction volume containing 2 µl each of 10 µM forward and 5' biotinylated reverse primers, 0.8 µl of the 10 mM deoxynucleotide trisphosphate mix dATP, dTTP, dCTP, dGTP, 5 µl of the 10x RedTaq polymerase reaction buffer stock solution (Sigma-Aldrich), and 1 µl of RedTaq polymerase (5 U/µl) in a Peltier thermal cycler PTC200 (BiozymDiagnostik, Hess. Oldendorf/Germany). For PCR of other genes, unbiotinylated reverse primers were used. Primers are given in STable 1. According to Fakhrai-Rad [2], the PCR product was immobilized by addition of 30 µl aqua bidest to 10 µl of PCR product, onto which 40 µl of binding buffer/beads mix were added and then incubated for 5 min at 24°C at 14000 rpm. Next, primer annealing was performed in a pyrosequencing plate whereby 12 µl of 1X annealing buffer and 3 µl of sequencing primer (STable 1) were added. The plate was then placed onto a vacuum prep tool for 5-7 seconds in 70 % ethanol, in denaturation solution (sodium hydroxide), followed by washing buffer and then incubated for 2 min at 80°C. While the plate cooled, dATPs, dTTPs, dGTPs and dCTPs were diluted 1:2 in 1X TE buffer pH 8.0 and pipetted into the pyrosequencing nucleotide dispensing tips. They were then placed in the respective position in the dispensing tip holder, along with the substrates (adenosine 5'phosphosulfate and luciferin) and enzymes needed (DNA polymerase, ATP sulfurylase, luciferase and apyrase). The plate was then placed into the pyrosequencer and the program was run as recommended.

1. Neufang G, Furstenberger G, Heidt M, *et al.* Abnormal differentiation of epidermis in transgenic mice constitutively expressing cyclooxygenase-2 in skin. *Proceedings of the National Academy of Sciences of the United States of America* 2001; **98**: 7629-7634.

2. Fakhrai-Rad H, Pourmand N, Ronaghi M. Pyrosequencing: an accurate detection platform for single nucleotide polymorphisms. *Human mutation* 2002; **19**: 479-485.

Supplementary Tables

STable 1: Primers, for genomic PCR, pyrosequencing, and qPCR

Gene	Primer Sequence
PCR (genomic mouse) and pyrosequencing	
K-Ras^{wt}	5'-GTCGACAAGCTCATGCGGGTG -3'<br>5'-CCTTTACAAGCGCACGCAGACTGTAGA-3'
K-Ras^{G12D}	5'-AGCTAGCCACCATGGCTTGAGTAAGTCTGCA-3'<br>5'-CCTTTACAAGCGCACGCAGACTGTAGA-3'
Cre	5'-ACCAGCCAGCTATCAACTCG-3'<br>5'-TTACATTGGTCCAGCCACC-3'
P48	5'-CTAGGCCACAGAATTGAAAGATCT-3'<br>5'-GTAGGTGGAAATTCTAGCATCATCC-3'
COX-2	5'-CCTAGATAACAGAGCCGCTTTC-3'<br>5'-TTTCACCATAGAATCCAGTCCG-3'
K-Ras ^{codons12,13}	
**Amplification:**	5'-GGCCTGCTGAAAATGACTGA-3'<br>Biotin-5'-TTAGCTGTATCGTCAAGGCGCTCT-3'
**Sequencing:**	5'-TGTGGTGGTTGGAGCT-3'
K-Ras^{codon61}	
**Amplification:**	5'-GACTCCTACAGGAAACAAG-3'<br>Biotin-5'-CTATAATGGTGAATATCTTC-3'
**Sequencing:**	5'-ATATTCTCGACACAGCAG-3'
qPCR (mouse)	
COX-1	5'-TGCATGTGGCTGTGGATGTCATCAA-3'<br>5'-CACTAAGACAGACCCGTCATCTCCA-3'
COX-2	5'-ACTCACTCAGTTTGTTGAGTCATTC-3'<br>5'-TTTGATTAGTACTGTAGGGTTAATG-3'
COX-2-3'UTR	5'-ATTCCTGCAGCCCAACATCTGT-3'<br>5'-AGAGGAGAAAACTTGGCTTGA-3'
Notch1	5'-CCTCAGATGGTGCTCTGATG-3'<br>5'-CTCAGGTCAGGGAGAACTAC-3'
Hey1	5'-TGAGCTGAGAAGGCTGGTAC-3'<br>5'-ACCCCAAACTCCGATAGTC-3'

Hes1	5'-GTCATCAAAGCCTATCATGGAG-3'<br>5'-GTGCGCCTGCCCGGGGTAGGTC-3'
Dll1	5'-CATCCGATACCCAGGTTGTC-3'<br>5'-ACGGCTTATGGTGAGTACAG-3'
qPCR (human)	
COX-1	5'-TGCCCAGCTCCTGGCCCGCCGCTT-3'<br>5'-GTGCATCAACACAGGCGCCTCTTC-3'
Notch-1	5'-CTCACCTGGTGCAGACCCAG-3'<br>5'-GCACCTGTAGCTGGTGGCTG-3'
Hes1	5'-TGG AAATGACAGTGAAGCACCT-3'<br>5'-GTTCATGCACTCGCTGAAGC -3'
Dll1	5'-CTACACGGGCAGGAA CTGCAG -3'<br>5'-CGCCTTCTTGTTGGTGTTCTTG-3'

STable 2

KEGG Pathways	P value	Genes
Downregulated Genes		
**Apoptosis**	0.0005	*CASP6, CSF2RB, IL1B, IL3RA, IRAK3, IRAK4, MAP3K14, PPP3R1, PRKAR1A, TNF*
**p53 Signaling**	0.010	*APAF1, ATM, BAI1, BAX, BBC3, CCND2, CCNG1, CDKN2A, SESN2, ZMAT3*
**Adherens Junction**	0.011	*ACTB, ACTN4, BAIAP2, NLK, PTPN6, PVRL1, PVRL4, SORBS1, SRC, TGFBR2, WASF2*
**Regulation of Actin Cytoskeleton**	0.037	*ACTB, ACTN4, ARHGEF1, ARPC1A, BAIAP2, DOCK1, FGF1, FGF13, FGF14, FGFR2, FGFR4, ITGA2B, ITGAE, ITGAM, ITGB1, MRAS, PFN1, PFN4, PIP4K2A, PIP5K1A, PPP1CA, RRAS, SRC, WASF2*
Upregulated Genes		
**Cell Cycle**	0.0082	*ANAPC1, ANAPC11, BUB3, CCNB1, CDC16, CHEK2, CUL1, E2F4, GADD45G, MCM4, MCM5, MCM6, MYC, PKMYT1, PLK1, RAD21, RB1, SMC3, STAG2, TTK, YWHAE, YWHAZ*
**MAPK Signaling**	0.0092	*ARRB1, ATF2, ATF4, BDNF, CACNA1D, CDC42, CHUK, DUSP1, DUSP16, DUSP6, DUSP9, EGFR, GADD45G, HSPA1A, JUN, KRAS, MAP3K12, MAPK7, MYC, PAK1, PLA2G4A, PLA2G4E, PRKACA, RAP1A, RASGRF1, RASGRP1, RELA, SOS1, STMN1, TRAF2*
**Pathways in Cancer**	0.012	*APC, BIRC3, CASP8, CDC42, CHUK, COL4A4, CTNNA2, CYCS, EGFR, FZD5, GSTP1, HSP90AA1, HSP90B1, ITGA3, JUN, KRAS, LAMA1, MSH2, MSH6, MYC, PIK3CB, PLCG2, PLD1, PPARG, PTCH2, RALA, RALBP1, RB1, RELA, RUNX1, SLC2A1, SOS1, STAT5B, TCF7L2,TRAF2, TRAF4,WNT3A*
**Mismatch Repair**	0.024	*EXO1, LIG1, MSH2, MSH6, POLD3*
**Pancreatic Cancer**	0.033	*ACDC42, CHUK, EGFR, KRAS, PIK3CB, PLD1, RALA, RALBP1, RB1, RELA*

STable 3

KEGG Pathways *(p≤0.005)*	P value	Genes
Upregulated Genes		
**Peroxisome**	0.0004	*ACSL4, ACSL5, CAT, MLYCD, MPV17L*
**Notch Signaling**	0.0005	ADAM17, MFNG, MYOD
**Glycerophospholipid Metabolism**	0.0063	*CEPT1, CRLS1, LCAT, PLA2G4E*
**Protein Processing in Endoplasmic Reticulum**	0.010	*HSPA1A, HYOU1, P4HB, PDIA6, PREB*
**P53 Signaling**	0.017	*GADD45G, RRM2, TNFRSF10B*
**Apoptosis**	0.034	*CEPT1, CRLS1, LCAT, PLA2G4E*
**Lipid Metabolism**	0.046	*CEPT1, PLA2G4E*
**Fatty Acid Metabolism**	0.050	*ACSL4, ACSL5*

Supplementary Figure Legends

SFig. 1: Genotyping of pancreatic DNA and pyrogram of K-Ras cancer-relevant codons
(a) To confirm the presence of the various constructs of interest (K5 COX-2, Cre-recombinase, and K-Ras$^{G12D/wt}$), in addition to the internal controls P48 promoter and endogenous K-Ras, genotyping for mice of interest was performed by PCR using 50 ng of DNA. **(b)** Pyrogram of a K5 COX-2$^{+/wt}$ mouse shows wild-type sequences in codons 12 (GGT), and 13 (GGC). **(c)** Pyrogram of a CPK mouse confirming the expected mutation in codon 12 (GAT), while harboring wild-type sequences in codon 13 (GGC).

SFig. 2: **Histology of 12 months-old C57BL6/N wild-type and C57BL6/N. K5 COX-2 675$^{+/wt}$ epithelial tissues.** Representative pictures of H&E-stained cryosections (n=4) from tail skin (a, b), urinary baldder (c, d), mammary gland (e, f), and pancreas (g, h) of wild type (a, c, e, g) and transgenic (b, d, f, h) mice. Note the epithelial hyperplastic phenotype in the transgenic epidermis, urinary bladder, and mammary gland (b, d, f) but not in pancreas (h). (d=duct, a=acinar tissue, v=blood vessel) Magnifications: 40x

SFig. 3: Histological analysis of pancreata from 12 months-old control cohorts. HE-stained sections show normal phenotypic appearance of monolayered ducts, abundant acinar parenchyma, Langerhans islets, as well as blood vessels in **(a)** WT, **(b)** P48-Cre, **(c)** K-Ras$^{G12D/wt}$, **(d)** K5 COX-2$^{+/wt}$, **(e)** P48-Cre.K5 COX-2$^{+/wt}$, and **(f)** K-Ras^{G12D}.K5 COX-2$^{+/wt}$ mice. Magnifications: 10x.

SFig. 4: Cytokeratin 5 (CK5) expression in pancreatic duct lesions of K5 COX-2$^{+/wt}$ **mice.** Indirect immunofluorescence staining using anti CK5-specific and anti-COX-2-specific antibodies and Cy3- and Alexa Fluor 488-coupled secondary antibodies, respectively. Note some CK5-positive ducts (red) and COX-2-positive cells (green) colocalizing with CK5-positive cells appearing in yellow in the merged figure. In addition, COX-2-positive cells come up in CK5-negative cells, indicating upregulation of endogeneous COX-2. The figure is representative for C/CP/CK mice. Nuclei were stained with Hoechst dye in blue. Magnifications: 63x.

SFig. 5: Nuclear expression of Ki67 proliferation marker in pancreatic ducts. Indirect immunofluorescence staining of WT/P/K (n=3), C/CP/CK (n=3), PK (n=3), and CPK (n=5) mice with Cy3-coupled secondary antibodies. Nuclei were stained in blue with Hoechst dye. Magnifications: 40x. Mean values $\pm$ SEM are given for Ki67 positive duct cells counted in the shown mouse cohorts (*$p \leq 0.05$, **$p<0.01$; ***$p<0.001$).

SFig. 6: Laser capture microdissection. Representative photomicrographs of pancreatic lesions selected before and after laser capture microdissection.

SFig. 7: Characterization of a primary cell culture generated from K5 COX-2 transgenic lesional pancreatic cysts. (a) RT-PCR of of total RNA extracted from a primary cell culture derived from K5 COX-2 transgenic pancreas (cl), wild-type (wt), as well as transgenic lesional (tr l) pancreas. Amplifation was performed using specific intron-spanning primers designed for amplification of transgenic and endogenous (tr+e) COX-2, transgenic (tr) COX-2-3'UTR, cytokeratin 19 (CK19), carbonic anhydarse-II (CA-II), Hes-1, elastase and β-actin. **(b)** CK5 staining (red) shows cytoplasmic peri nuclear expression in pancreatic cells. Green signals observed in the cytoplasm and peri nuclear compartments reflect COX-2 expression. CK5 and COX-2 colocalization is illustrated by the yellowish merge observed. Hes-1

expression is limited to the nuclei of cells. Merge of Hes-1 with COX-2 signals shows colocalization in the same cell. Magnifications 40x

SFig. 8: Inhibition of Ras activation and its down-stream effector kinases in BxPC3 cells by celebrex. As compared to mock-treated BxPC3 cells (0)**,** treatment of cells with 20 μM celebrex for 48 hours reduces the levels of active GTP-bound Ras and phosphorylated forms of the effector kinases ERK and AKT, while the levels of the total Ras, ERK, and AKT remain unchanged. Beta actin is included as a loading control.

SFigure 1

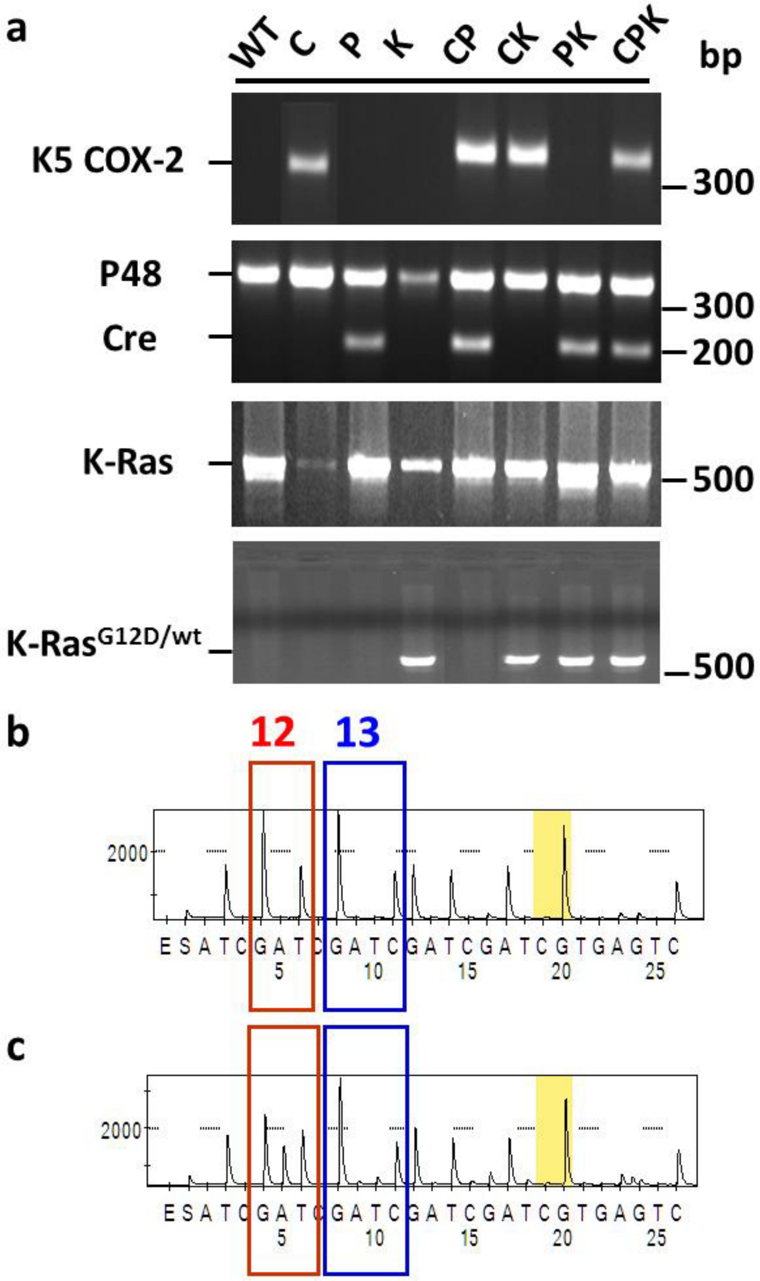

a
WT
C
P
K
CP
CK
PK
CPK
bp
K5 COX-2
300
P48
Cre
300
200
K-Ras
500
K-Ras G12D/wt
500
b
12
13
2000
E S A T C G A T C G A T C G A T C G A T C G T G A G T C
5
10
15
20
25
c
2000
E S A T C G A T C G A T C G A T C G A T C G T G A G T C
5
10
15
20
25

SFigure 2

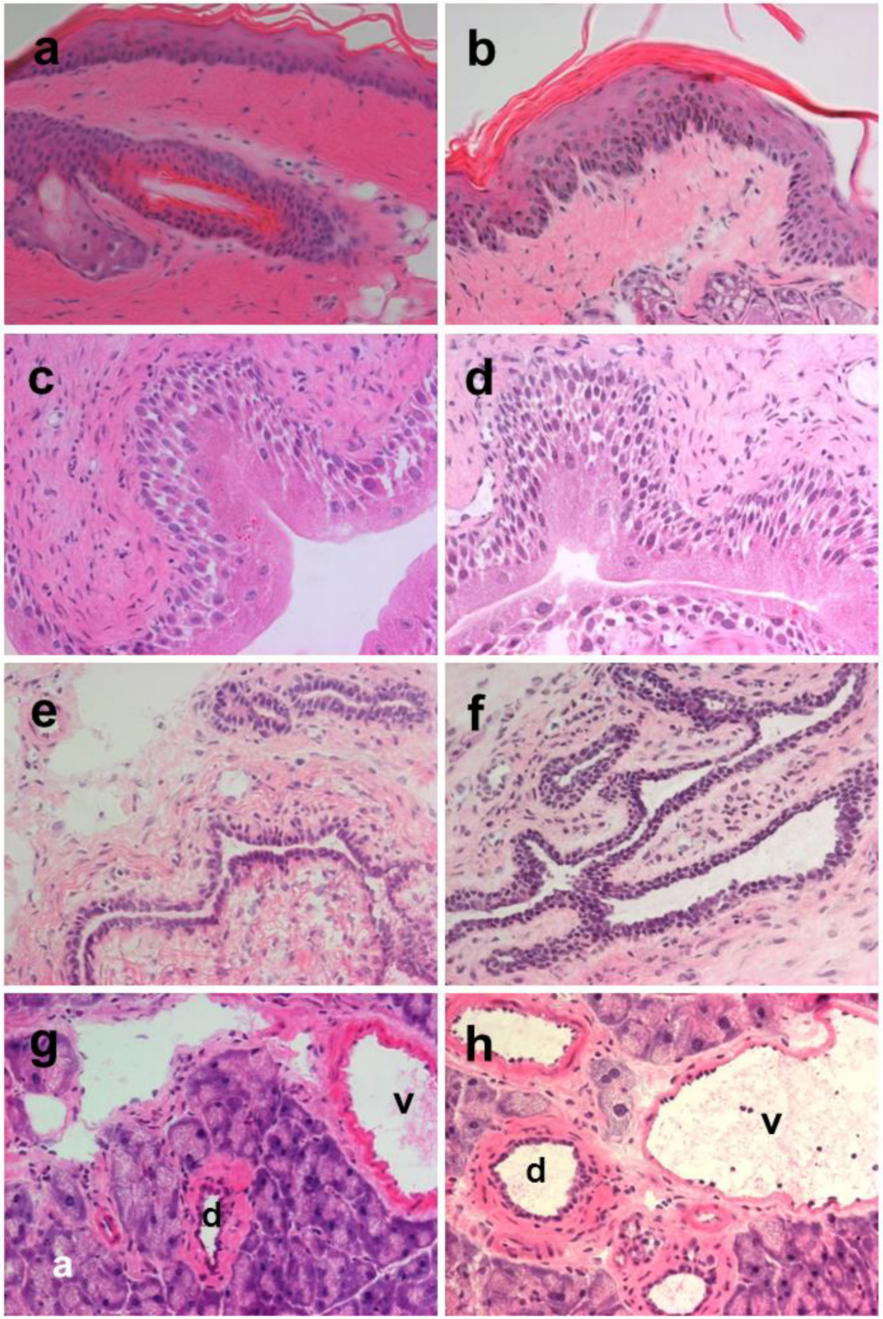

SFigure 3

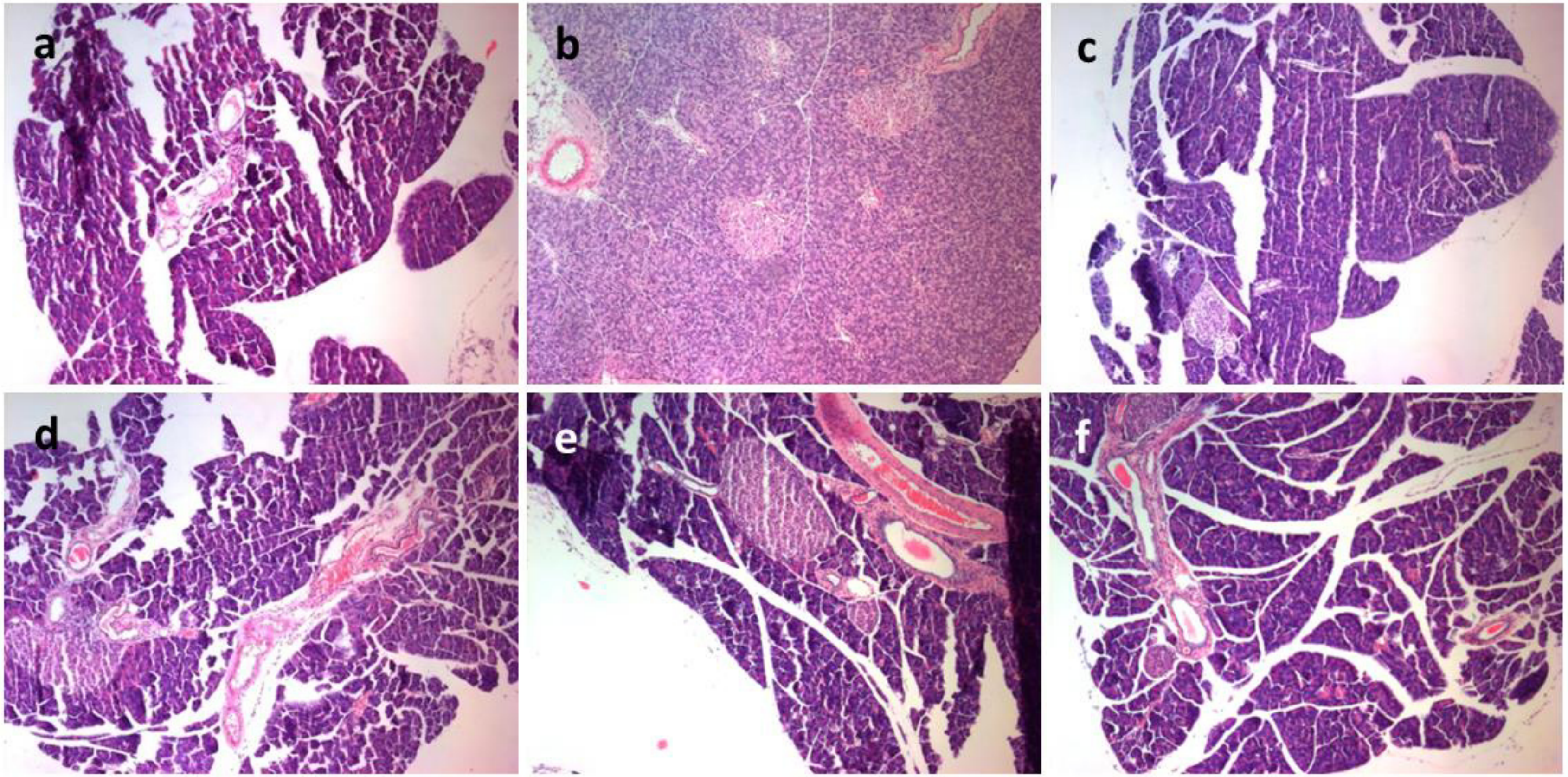

SFigure 4

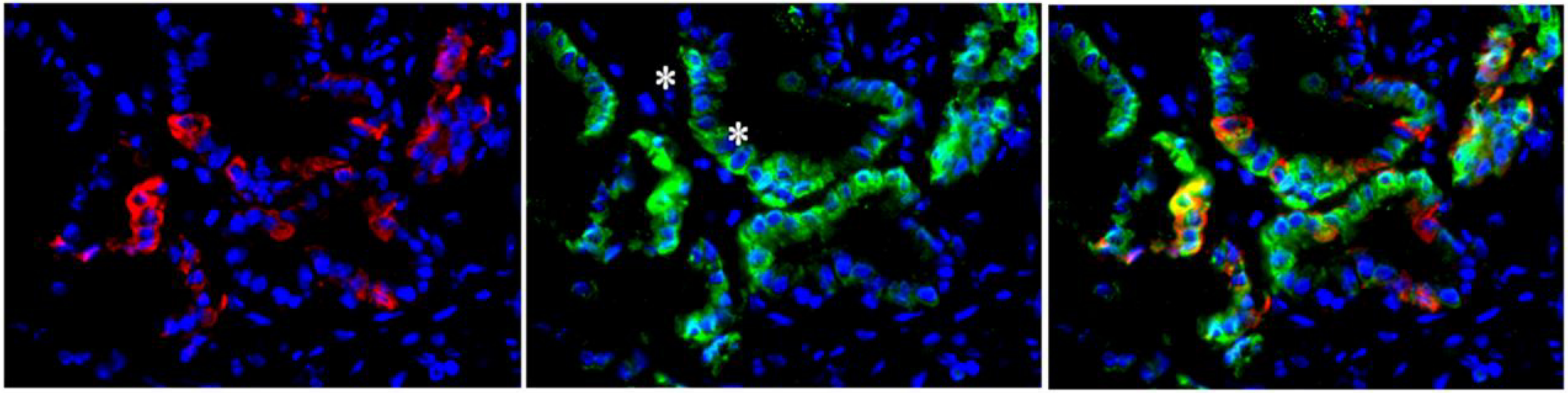

keratin 5
nuclei /Hoechst

COX-2

merged

SFigure 5

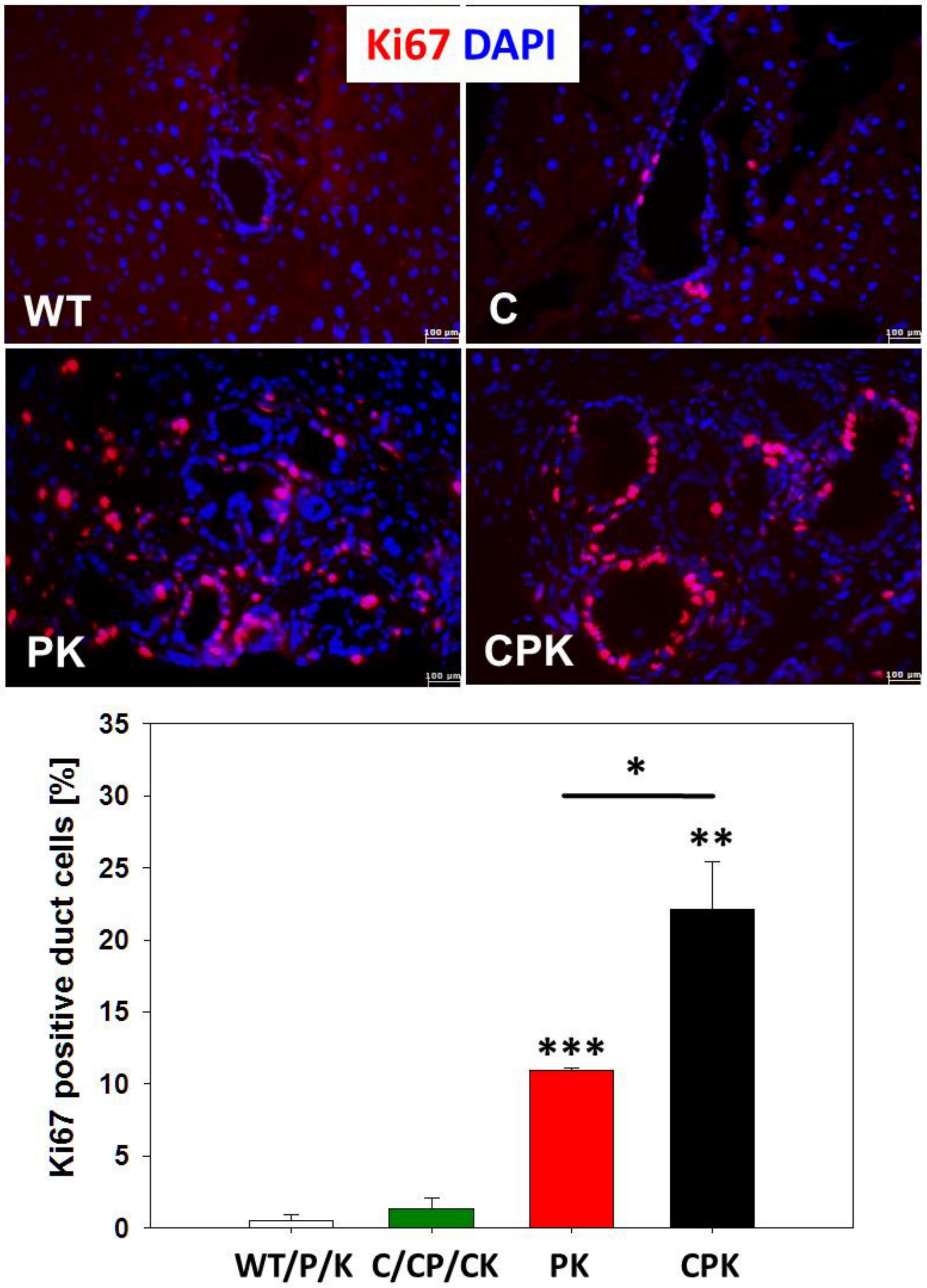

Ki67 DAPI
WT
C
PK
CPK
35
30
25
20
15
10
5
0
Ki67 positive duct cells [%]
*
**
***
WT/P/K
C/CP/CK
PK
CPK

SFigure 6

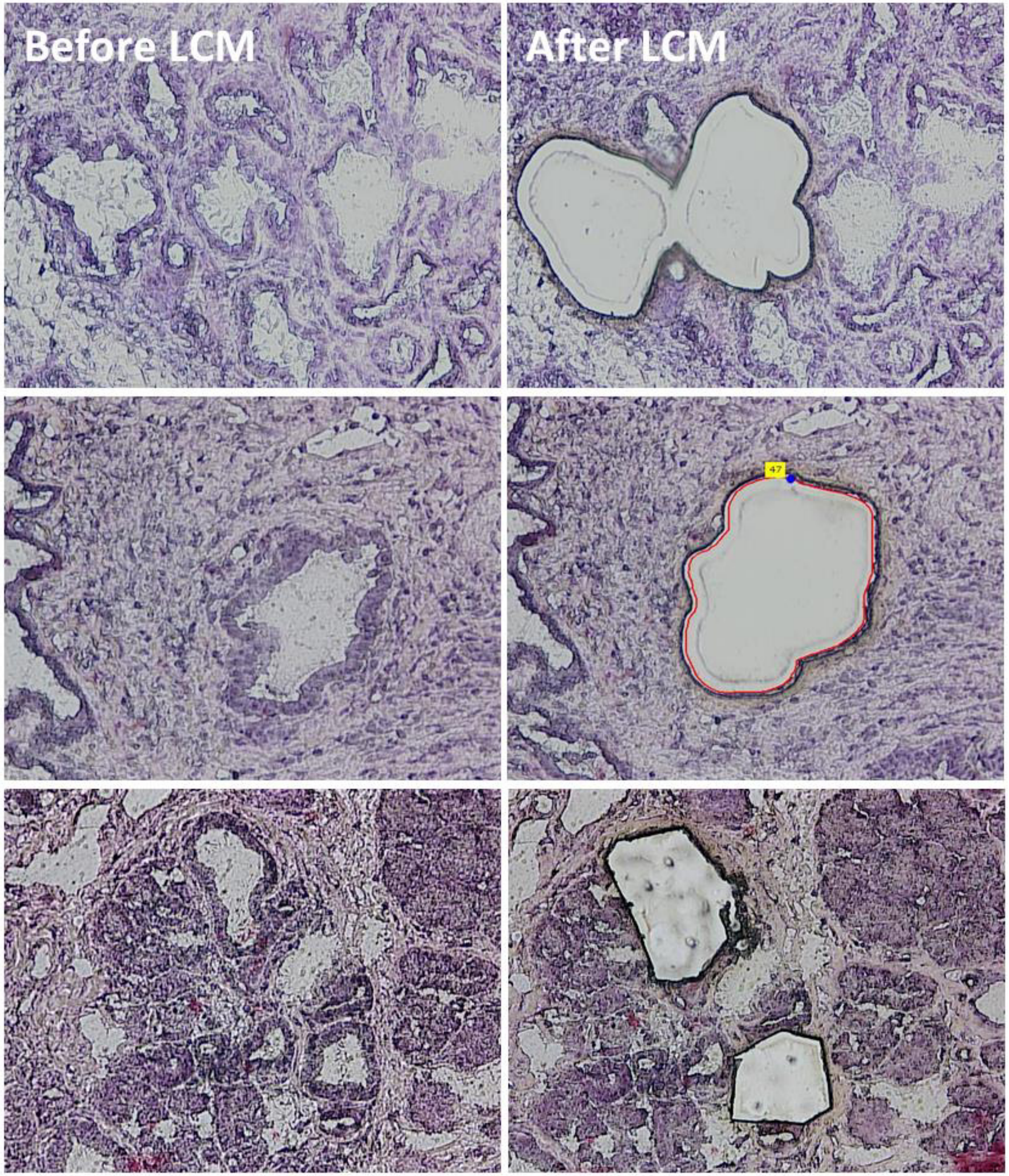

SFigure 7

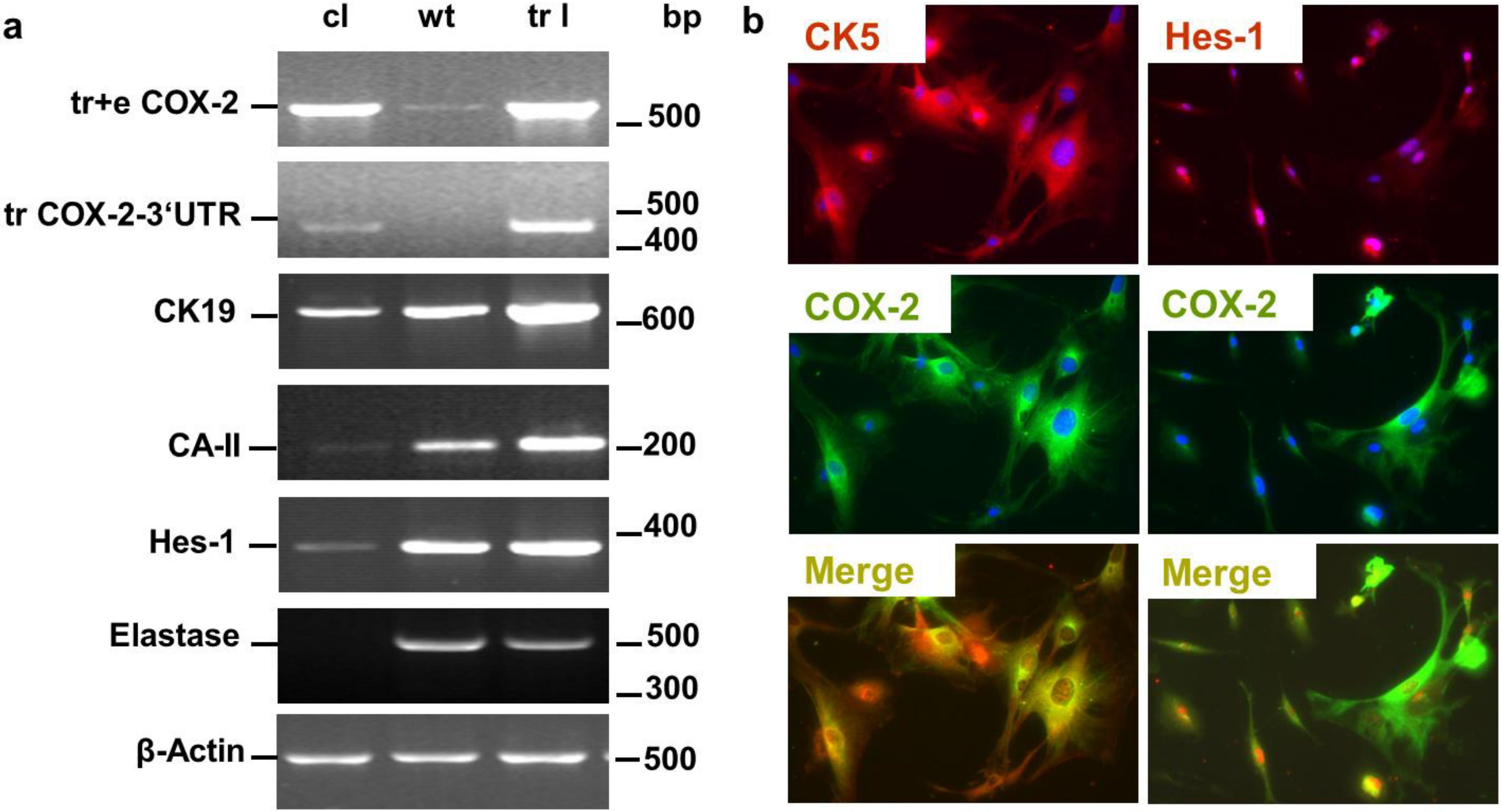
a
cl
wt
tr l
bp
tr+e COX-2
500
tr COX-2-3‘UTR
500
400
CK19
600
CA-II
200
Hes-1
400
Elastase
500
300
β-Actin
500
b
CK5
Hes-1
COX-2
COX-2
Merge
Merge

SFigure 8

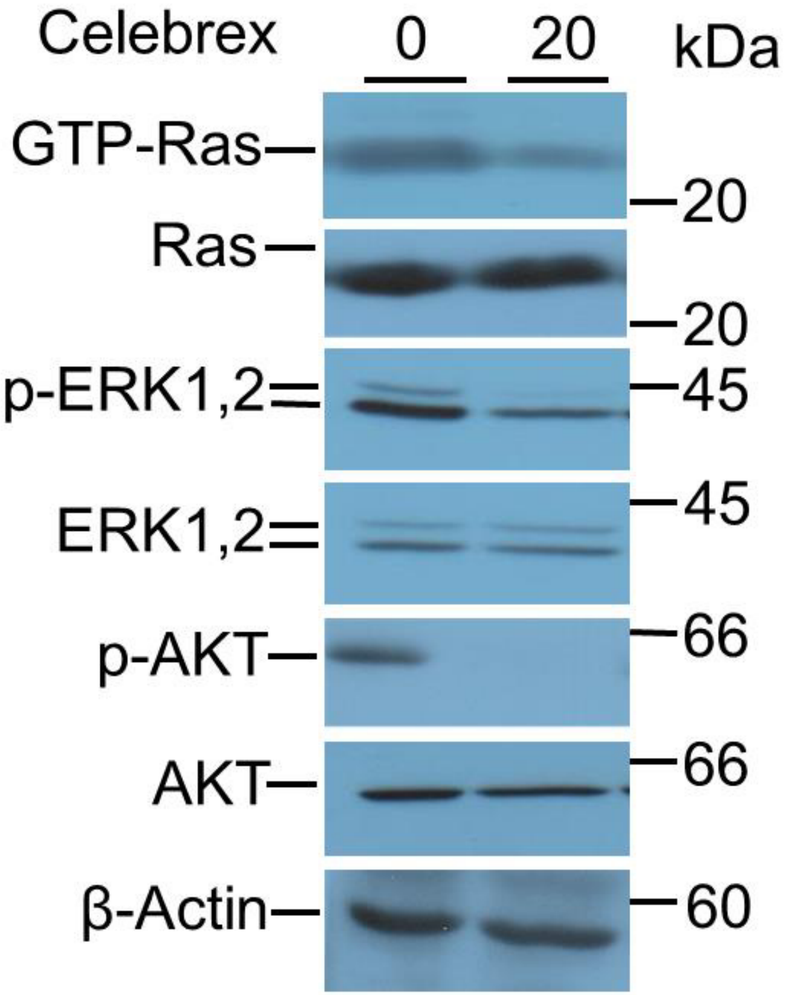
Celebrex
0
20
kDa
GTP-Ras
20
Ras
20
p-ERK1,2
45
ERK1,2
45
p-AKT
66
AKT
66
β-Actin
60
